# Insertion of an extra copy of Xq22.2 into 1p36 results in functional duplication of the *PLP1* gene in a girl with classical Pelizaeus-Merzbacher disease

**DOI:** 10.1186/s12881-015-0226-6

**Published:** 2015-09-02

**Authors:** Julien Masliah-Planchon, Céline Dupont, George Vartzelis, Aurélien Trimouille, Eléonore Eymard-Pierre, Mathilde Gay-Bellile, Florence Renaldo, Imen Dorboz, Cécile Pagan, Samuel Quentin, Monique Elmaleh, Christina Kotsogianni, Elissavet Konstantelou, Séverine Drunat, Anne-Claude Tabet, Odile Boespflug-Tanguy

**Affiliations:** UF de Génétique moléculaire, Hôpital Robert Debré, AP-HP, Paris, France; Inserm U1141, Université Paris Diderot, Sorbonne Paris Cité, Hôpital Robert Debré, Paris, France; New adresse: Unité de Génétique Somatique, Institut Curie, Paris, France; UF de Cytogénétique, Hôpital Robert Debré, AP-HP, Paris, France; Paediatric Neurology, P&A Kyriakou Paed. Hospital, Athens Medical School, Athens, Greece; Cytogénétique Médicale, Univ Clermont1, UFR Médecine, CHU Estaing, Clermont-Ferrand, France; ERTICa, EA 4677, Univ Clermont1, UFR Médecine, Clermont-Ferrand, France; Neurologie et maladie métabolique, Hôpital Robert Debré, AP-HP, Paris, France; IUH, Hôpital Saint-Louis, Paris, France; Imagerie pédiatrique, Hôpital Robert Debré, AP-HP, Paris, France

## Abstract

**Background:**

Pelizaeus-Merzbacher disease (PMD) is an X-linked dysmyelinating disorder characterized by nystagmus, hypotonia, ataxia, progressive spasticity, and cognitive decline. PMD classically results from a duplication of a genomic segment encompassing the entire *PLP1* gene. Since the *PLP1* gene is located in Xq22, PMD affects mostly boys.

**Methods and results:**

Here we report the case of a girl with typical PMD. Copy number analysis of the *PLP1* locus revealed a duplication of the entire gene and FISH analysis showed that the extra copy of the *PLP1* gene was actually inserted in chromosome 1p36. This insertion of an additional copy of *PLP1* in an autosome led to a functional duplication irrespective of the X-inactivation pattern. Subsequent overexpression of *PLP1* was the cause of the PMD phenotype observed in this girl. Further sequencing of the breakpoint junction revealed a microhomology and thus suggested a replication based mechanism (such as FoSTeS or MMBIR).

**Conclusion:**

This case emphasizes the susceptibility of the *PLP1* locus to complex rearrangement likely driven by the Xq22 local genomic architecture. In addition, careful consideration should be given to girls with classical PMD clinical features since they usually experience complex *PLP1* genomic alteration with a distinct risk of inheritance.

## Background

Pelizaeus-Merzbacher disease (PMD; MIM#312080) is a rare X-linked hypomyelinating leukodystrophy related to genomic alterations of the proteolipid protein 1 (*PLP1*) gene [1]. *PLP1* encodes both major proteins of the central nervous system (CNS) myelin, PLP and DM20. PMD is characterized by early nystagmus, hypotonia and ataxia with subsequent signs of neurodegeneration characterized by severe spasticity and cognitive impairment. However, a large clinical spectrum exists from severe forms without motor acquisitions and dystonia (PMD0), to mild forms with acquired walking capacities (PMD3), and the mildest form of PLP-pathies, spastic paraplegia type 2 (SPG2, MIM#312920). An obvious genotype-phenotype correlation has been described in PMD. Missense mutations are rare but usually cause the most severe forms, whereas *PLP1* loss-of-function alterations (null mutations and large deletions) lead to the mildest PMD/SPG2 forms. Classical PMD is commonly caused by duplication of a genomic segment containing the entire *PLP1* gene. *PLP1* gene duplication results in PLP overexpression and subsequent accumulation in the cytoplasm of the myelinating oligodendrocytes leading to cellular stress and CNS myelin defect. A gain of three to five copies of the *PLP1* gene have been rarely described and are associated with a severe phenotype [2, 3]. PMD affects almost exclusively boys and most females who carry and transmit *PLP1* genomic alterations to their affected sons are asymptomatic or manifest mild, late-onset spastic paraplegia or cognitive impairment. Few cases of girls presenting a phenotype of PMD in childhood have been described. Foncesca et al. reported the case of a constitutional translocation t(X;22)(q22;q13) with an additional *PLP1* copy at the breakpoint region in a girl with classical PMD [4]. Yiu et al. reported another case of a PMD girl with an unbalanced chromosomal translocation of *PLP1* into 1p36 [5]. Additional cases of girls with PMD due to very large *PLP1* gene duplications have been reported [6].

Here we report the case of a 2 year old girl with typical PMD who has an insertion of an extra copy of a genomic segment of Xq22 containing the entire *PLP1* gene into chromosome 1p36. This insertion of an additional active copy of *PLP1* in an autosome led to a functional duplication. Further delineation of the breakpoint junction sequence revealed a microhomology and thus support a replication based mechanism such as FoSTeS and MMBIR. Our result further emphasizes the large diversity of the genomic rearrangement involving the *PLP1* gene probably based on the complexity of the genomic architecture [7].

## Methods

### Multiplex ligation-dependent probe amplification (MLPA)

MLPA was conducted according to the supplied manufacturer’s specifications using the probemix P022-PLP1 (MRC-holland, Amsterdam, Holland). PCR products were then mixed with formamide (HiDi Formamide, Applied Biosystems, Foster City, CA) and fluorescent Genescan 500 LIZ size standard (Applied Biosystems) prior to analysis using an Applied Biosystems ABI 3130xl capillary sequencer (Applied Biosystems). Data collection and export used GeneMapper software (Applied Biosystems). Peak areas of each fragment were compared to those of a control sample to calculate the gene dosage of each amplicon, including those corresponding to each exon of the *PLP1* gene.

### X chromosome inactivation pattern

The analysis of the X inactivation pattern was based on extend human androgen receptor (HUMARA) assay proposed Bertelsen et al. [8]. Briefly, 200 ng of leukocyte DNA was digested with HpaII (New England Biolabs, Ipswich, MA) at 37 °C for 12 h. PCR amplification of four loci (*AR*, *PCSK1N*, *SLITRK4*, and *ZDHHC15*) was performed on undigested DNA and HpaII digested DNA. PCR products were subsequently analyzed on an ABI 3130xl Genetic Analyzer (Applied Biosystems) using GeneMapper software (Applied Biosystems). The X-inactivation ratios were calculated as previously described and skewed X chromosome inactivation was considered if this ratio decrease below 20 % in the investigated blood sample [8].

### *PLP1* gene expression analysis in patient fibroblasts

Total RNA was isolated from fibroblasts of eight controls without copy number variation of the *PLP1* gene, of one positive control with a duplication of *PLP1*, and of our patient using the guanidinium thiocyanate-phenol-chloroform method. Ten microgram was used to generate cDNA using Superscript II first-strand cDNA synthesis kit (Invitrogen, Carlsbad, CA, USA) with oligo (dT) primers, according to the manufacturer’s protocol. Our patient and control cDNAs were used for *PLP1* gene expression analysis performed by real-time PCR according to previously reported conditions [9]. Specifically designed primers were used to determined total *PLP1* gene expression after normalization to the beta-glucuronidase gene (*GUSB*) expression using the ΔΔCt method.

### Microarray

Creation of a custom array CGH design was performed using the Agilent software eArray (https://earray.chem.agilent.com/earray/), referred to the GRCh37/hg19 assembly to cover the *PLP1* genomic region with maximum resolution. The microarray contained a total of 30,032 probes spanning a 20 Mb region surrounding the *PLP1* gene (Agilent Technologies, Santa Clara, CA). Those 30,032 probes were distributed between three groups with various probe densities. The first group includes 15,032 probes encompassing 2 Mb (chrX: 102,000,000–104,000,000; corresponding to a 150 bp average probe spacing) around the *PLP1* gene and includes most of the *PLP1* duplications described so far [10, 11]. A second, largest interval contained 10,000 probes surrounding 8 Mb (chrX: 98,000,000–102,000,000 and chrX: 104,000,000–106,000,000; 800 bp average probe spacing). The last interval was designed to encompass the largest *PLP1* rearrangement and includes 5,000 probes spanning 10 Mb (chrX: 93,000,000–98,000,000 and chrX: 106,000,000–113,000,000; 2000 bp average probe spacing) [6].

A HumanCytoSNP-12 (V2.1) pangenomic microarray (Illumina, San Diego, CA USA) was also performed.

### Fluorescence in situ hybridization (FISH)

Fluorescence in situ hybridization (FISH) was performed on lymphocytes metaphase spreads according to standard protocols. FISH analysis was carried out with the probe RP11-832L2 spanning 183 Kb (chrX:102,902,650–103,085,315), which encompasses the entire *PLP1* gene, together with a subtelomeric probe of the short arm of chromosome 1 (CEB108/T7).

### Genome walker

The Universal Genome Walking Kit (Clontech, Palo Alto, CA, USA) was used to identify precise breakpoints of the inserted *PLP1*-containing X chromosome segment into 1p36. According to the manufacturer’s protocol, the genomic DNA of the patient was completely digested with four separate restriction enzymes (i.e. EcoRV, DraI, PvuII, and SspI) and subsequently ligated to the GenomeWalker adaptors. The GenomeWalker libraries produced were then used as a template for primary PCR amplification. The primers used were the outer adaptor primer provided in the kit and a Xq22 specific primer localized at the boundaries of the duplicated *PLP1* segment identified with our highly resolutive PLP1-custom array CGH. A secondary nested PCR using the nested adaptor primer and a nested Xq22 specific primer was then performed. Sequencing of the PCR products was finally achieved if a single or two (considering that both, the normal Xq22 and the 1p36 derivative, alleles could be digested and ligated to the adaptor) specific and major PCR products were visible after electrophoresis through an agarose gel stained with GelRed (Biotium, Hayward, CA, USA). Sequencing was conducted using the nested primers with the BigDye terminator sequencing kit (Applied Biosystems).

## Results

### Case report

**Fig. 1:**
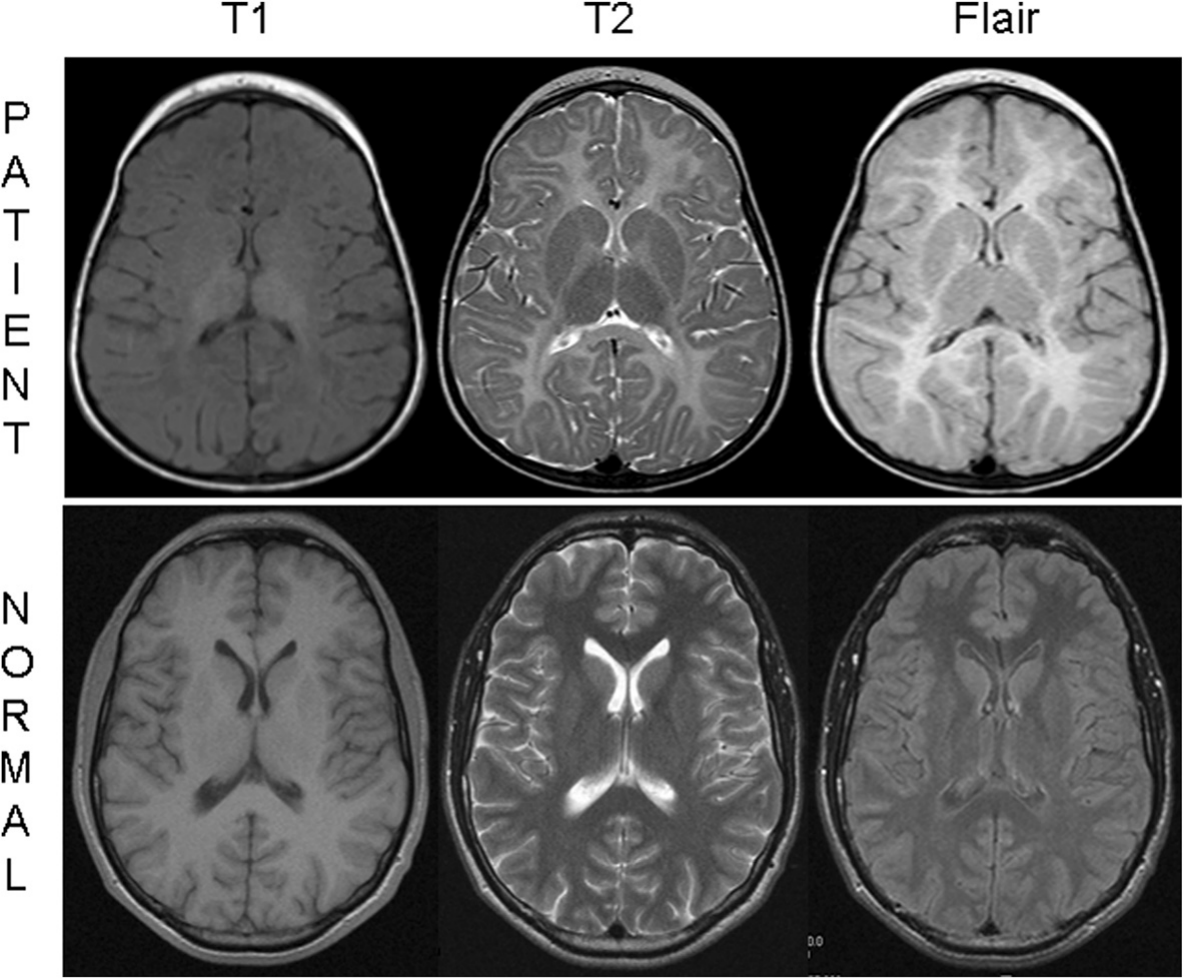
Patient’s brain magnetic resonance imaging (MRI). Sagittal T1-weighted MRI showed iso/hyposignal of the whole cerebral white matter, axial T2-weighted, and FLAIR images showing diffuse hypersignal of the hemispheric white matter, internal capsule, and corpus callosum in the patient at 2.5 years compared to an age-matched healthy child

Here we report the case of a girl who is the third child from unrelated and healthy parents of Greek origin. Pregnancy was uneventful, however during the second month of life a horizontal nystagmus and severe hypotonia were noticed. At 9 months of age, a brain magnetic resonance imaging (MRI) was performed due to a persistent delay in motor milestones associated with ataxia of the head and trunk led; MRI revealed a severe delay in the myelination process (evaluated at 2 months of age). Afterwards, she improved obviously in motor capacities (holding her head up at 12 months, sitting with support at 16 months, and standing position at 30 months) with excellent psychosocial interactions and decreased nystagmus intensity. Another brain MRI at 33 months of age confirmed the diffuse “hypomyelinating” pattern of the supratentorial cerebral white matter (Fig. 1). In addition, brainstem auditory evoked potentials showed an exclusive cochlear wave I without subsequent recordable brain waves suggesting a severe impairment in CNS conduction. No other abnormalities including dysmorphic features, heart, kidney, or liver problems were found. Now aged 4 years, she continues to improve (crawling, walking with support, feeding herself, drawing a line, speaking using comprehensive simple sentences). However, signs of pyramidal tract dysfunctions are clearly present in the lower limbs and MRI abnormalities have not changed. Those clinical features strongly suggested the diagnosis of PMD.

### Characterization of the *PLP1* gene duplication

MLPA analysis of the DNA extracted from the patient’s blood revealed a duplication of the seven probes localized in each coding exon of the *PLP1* gene. Additional probes that were duplicated revealed that the duplication extended at least from the *TCEAL1* to *ESX1* genes thus encompassing at least 614 Kb (but less than 1685 Kb).

**Fig. 2:**
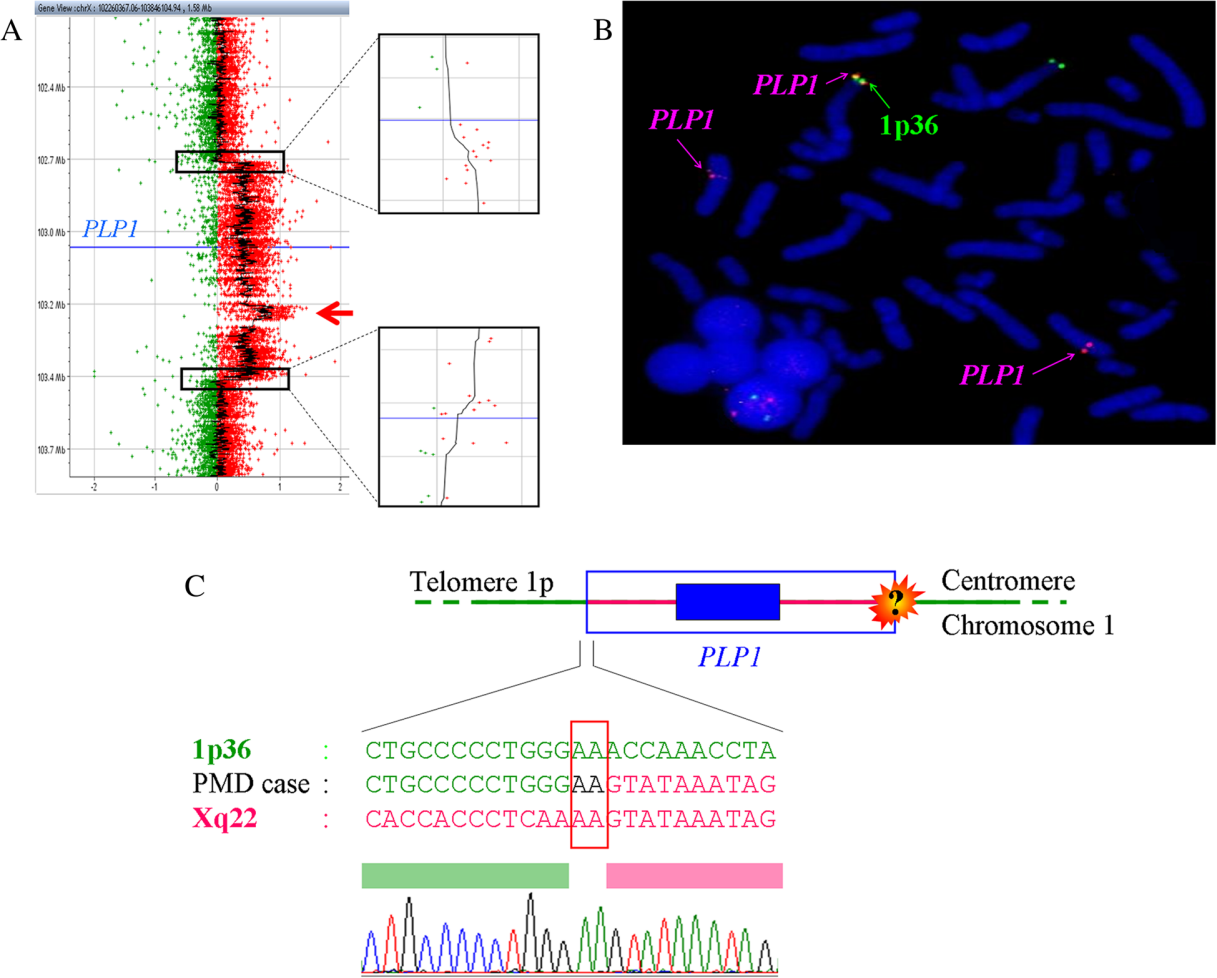
Insertion of an Xq22 segment including *PLP1* in 1p36. **a** Custom PLP1 array CGH profile confirms the *PLP1* gene (*centered on the blue bar*) duplication and precise the breakpoint of the duplicated genomic segment expanding from 102,761,000 to 103,513,000 (752 Kb of length) on Xq22 (the *red arrow* indicates the embedded triplicated segment). **b** FISH analysis with the RP11-832L2 PLP1-specific and the CEB108/T7 1p36 subtelomeric probes reveals that the additional *PLP1* copy is inserted into autosome 1p36. **c** Sequencing of a breakpoint of the insertion of *PLP1*-containing segment in 1p36 chromosome reveals a two-base pair microhomology

To confirm the *PLP1* duplication and define more precisely the size of the duplicated segment we used a custom array CGH (aCGH) designed to encompass the Xq22 genomic region containing the *PLP1* gene with maximum resolution. The aCGH profile confirmed the MLPA result and specified the breakpoint of the duplicated genomic segment covering from 102,761,000 to 103,513,000 (752 Kb of length) on Xq22 according to the GRCh37/hg19 assembly (Fig. 2a).

We next performed FISH analysis with a BAC probe specific to the *PLP1* locus (RP11-832L2, Xq22.2) on lymphocyte metaphase spreads. Three distinct *PLP1*-specific signals were visualized consistent with the *PLP1* duplication in a girl. Two probes bound to the normal Xq22 band of both X chromosomes together with an additional ectopic signal that bound just proximal to the CEB108/T7 1p36 subtelomeric probe (Fig. 2b). Another FISH analysis with BAC probes localized to X chromosome telomeres did not reveal a translocation involving the X chromosome. Hence, the additional copy of *PLP1* results from an insertion into chromosome 1p36 rather than a classical translocation. A whole genome microarray confirmed the *PLP1* duplication but did not reveal any copy number alteration in 1p36 region. MLPA analysis in the parents did not reveal any variation in *PLP1* copy number. However, we wondered if this rearrangement was nevertheless inherited from a parental balanced rearrangement. Therefore we performed the *PLP1* specific FISH analysis on the parents’ blood and did not detect any ectopic localization of the *PLP1* gene. We thus conclude that this rearrangement was acquired either in the gametes of one of the parents or *de novo*.

### Absence of skewed X inactivation pattern

A skewed X inactivation pattern resulting in inactivation of the X chromosome carrying the abnormal *PLP1* gene has been suggested to explain why most females who carry *PLP1* genomic alteration are asymptomatic [12]. We thus performed an analysis of the X inactivation pattern based on the extend HUMARA assay [8]. This analysis of DNA extracted from a blood sample from the patient did not reveal a significant skewed pattern for any of the four X chromosome loci explored (i.e. *AR*, *PCSK1N*, *SLITRK4*, and *ZDHHC15*). Although we cannot assert that there is not a skewed X chromosome inactivation pattern in the oligodendrocytes of the patient, the insertion of an extra-copy of *PLP1* in an autosome did lead to a functional duplication irrespective of the X-inactivation pattern.

### *PLP1* over-expression in patient fibroblasts

We also quantified the *PLP1* gene expression in fibroblasts from the patient and compared it to expression in normal control fibroblasts and fibroblasts from male patients with classical tandem *PLP1* duplications. The duplicated controls and our patient showed a 1.88 and a 5.21-fold increase of the *PLP1* expression respectively compared to the normal control. This result confirmed the functional duplication of the *PLP1* gene leading to its overexpression in the patient’s fibroblasts.

### Precise mapping of the breakpoint using genome walking strategy

To further characterize the insertion site of *PLP1* on chromosome 1p36, we performed a genome walking strategy. This molecular procedure allows sequencing of the 1p36 telomeric flanking segment directly adjacent to the breakpoint of the duplicated genomic region of Xq22 (Fig. 2c). The Xq22 segment inserted exactly at the position chr1:140,332 (GRCh37/hg19) on chromosome 1p36. In addition, we identified 2 bp of microhomology shared by the 1p36 and the Xq22 boundaries genomic sequences.

## Discussion

We have described the case of a girl with a classical form of PMD due to an insertion of the *PLP1* gene into 1p36 autosome. *PLP1* gene duplication in boys results in PLP overexpression and subsequent accumulation in the cytoplasm of the myelinating oligodendrocytes, leading to a CNS myelin defect. We demonstrated that the *PLP1* transcript is overexpressed in the fibroblasts of our patient with an insertion of an additional copy of *PLP1* in chromosome 1p36. It has been proposed that *PLP1* expression in fibroblasts is a good cellular model for reflecting the consequences of an altered *PLP1* gene dosage [9]. We thus assume that the additional copy of *PLP1* led to an overexpression of PLP in oligodendrocytes and that subsequent toxicity in this cell type was responsible for the neurological phenotype. The *PLP1* overexpression seems even higher in our patient’s fibroblasts compared to classical tandem duplications in boys. This could be related to the epigenetic state of the chromatin environment in the 1p36 insertion site that could be more favorable to gene expression than the Xq22 *PLP1* locus.

**Fig. 3:**
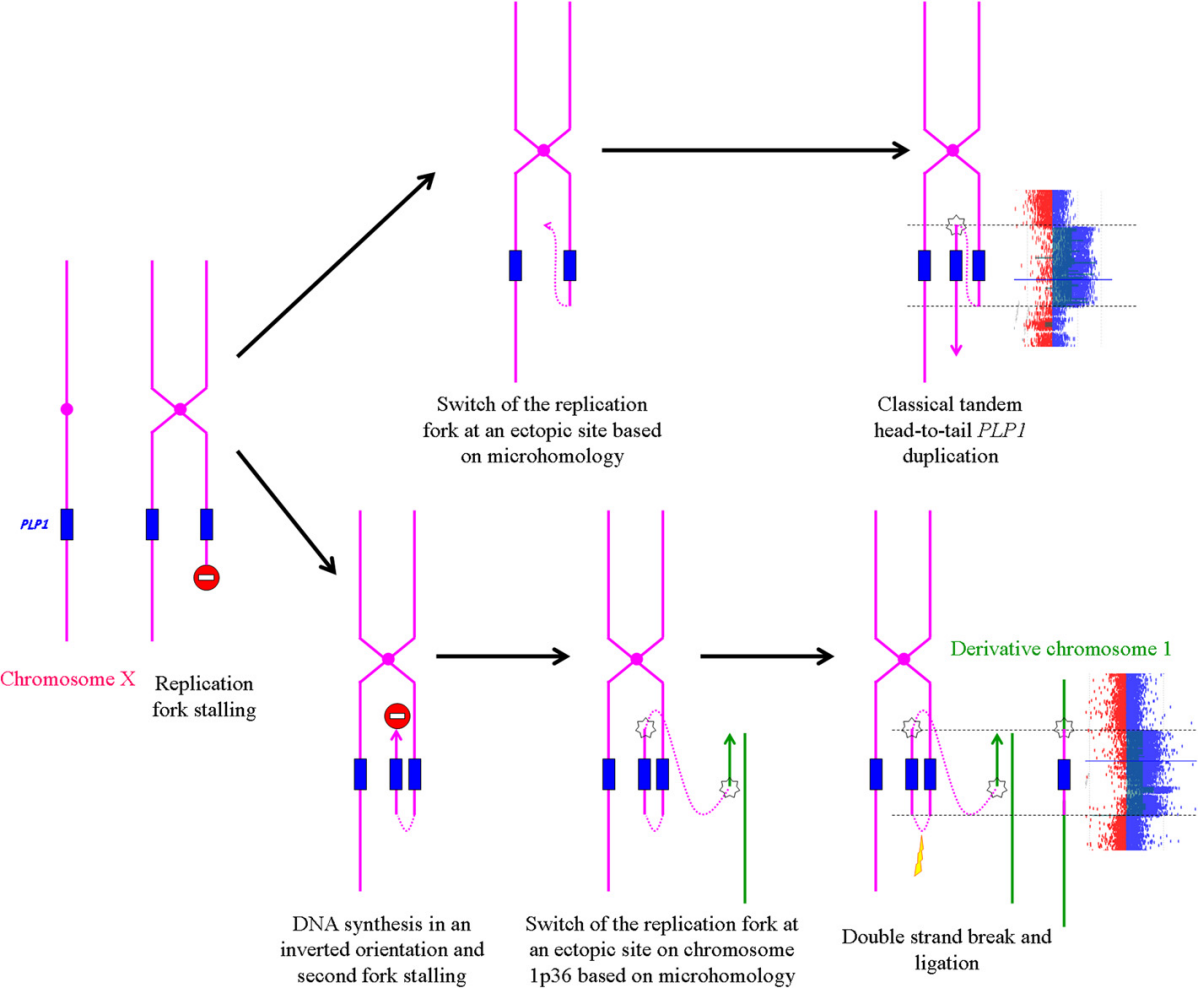
Hypothetical model of mechanism for the insertion of *PLP1* in 1p36. Based on the FoSTeS mechanism proposed by Lupski and coll. [10] (*upper situation*) we propose an alternative model to explain the insertion of an extra copy of the *PLP1* gene in chromosome 1p36 (*lower situation*)

Sequence analysis of the boundaries of the Xq22 duplicated segment revealed two nucleotides of microhomology at the telomeric breakpoint junction (Fig. 2c) consistent with a replication based mechanism, such as fork stalling and template switching (FoSTeS) [10] or microhomology-mediated break-induced replication (MMBIR) [13]. We cannot rule out that the two-base microhomology observed at the breakpoint occurs by chance and is not related to a replicative-based mechanism. However, it is noteworthy that James Lupski and colleagues firstly described the elegant FoSTeS mechanism precisely at the *PLP1* locus [10]. Based on this model, we propose a genomic mechanism to explain the microhomology-based insertion of *PLP1* in an autosome (Fig. 3): instead of switching to a template on the sister chromatid, as it has been classically described, the replication fork may switch to chromosome 1p36. Indeed, in the classical model (i.e. intrachomosomal rearrangement), both replication forks may be separated by large genomic sequence leading to extensive duplication or deletion. It is reasonable to believe that the new replication fork reestablish by considering the physical proximity of the new microhomology-based site. Thus, the new replication fork could take place on distinct chromosome if the insertion site is physically close considering the three-dimensional structure of the chromatin in interphase nuclei. This may be supported by the fact that another *PLP1* rearrangement involving the 1p36 genomic region has already been described [5].

Despite several primer combinations and PCR conditions we were unable to obtain the second breakpoint junction. The inability to obtain a PCR product for the centromeric breakpoint may be related to the complexity of the rearrangement at this breakpoint. Indeed, the *PLP1*-custom aCGH revealed a triplicated segment embedded in the *PLP1* duplication from chrX:103,230,500 to chrX:103,317,500 (Fig. 2a). Triplications of the *PLP1* gene have already been described and are associated with a more severe phenotype compared with patients with duplications [2, 3]. However, in our case, the triplicated segment did not include the *PLP1* gene. This is consistent with the mild phenotype of our patient compared to PMD boys with three copies of *PLP1*. It has been proposed that triplications at the *PLP1* locus result from a duplication-inverted triplication-duplication (DUP-TRP/INV-DUP) likely based on a replication based mechanism and that relies on the presence of segmental duplications in an inverted orientation [14]. Interestingly, the triplicated segment observed in our case occurred between two segmental duplications encompassing the *H2BFXP* pseudogenes localized 60 Kb from each other in an inverted direction. This triplicated segment further illustrates the complexity of the observed rearrangement.

## Conclusion

Here we describe the case of a girl with a classical form of PMD due to an insertion of a 752 Kb Xq22 region containing the whole *PLP1* gene into 1p36 autosome. This rearrangement harbors a two base-pair microhomology at one of the breakpoint suggesting a replication based mechanism such as FoSTeS or MMBIR. In addition to *PLP1* duplications, other genomic rearrangements responsible for PMD, including deletions [15, 16], and translocations [4] have been described. Complex genomic rearrangements including duplication-inverted triplications [2, 3] and complex duplications consisting of two duplicated fragments interspersed with a segment without copy number variation [6] have also been identified at the *PLP1* locus. Our result further emphasizes the large diversity of the genomic rearrangement involving the *PLP1* gene probably based on the complexity of the genomic architecture [7]. Finally, from a clinical point of view, careful consideration should be given to girls with classical PMD clinical features since they usually experience complex *PLP1* genomic alteration with distinct risk of inheritance. FISH analysis should thus be considered, in addition to copy number evaluation and *PLP1* gene sequencing, to detect those complex rearrangements in girls with PMD.
